# Reproducible measurements of the δ^2^H composition of non-exchangeable hydrogen in complex organic materials using the UniPrep2 online static vapour equilibration and sample drying system

**DOI:** 10.1016/j.mex.2022.101984

**Published:** 2023-01-04

**Authors:** Leonard I. Wassenaar, Leonardo Sisti, Matthias Pilecky, Martin Kainz

**Affiliations:** a WasserCluster Lunz - Biologische Station, Dr. Carl Kupelwieser Promenade 5, Lunz am See A-3293, Austria; b Eurovector C/O Polo Tecnologico di Pavia, Via F.lli Cuzio, 42, Pavia 27100, Italy; c Department of Biomedical Research, Donau-Universität Krems, Dr.-Karl-Dorrek-Strasse 30, Krems an der Donau A-3500, Austria

**Keywords:** Hydrogen isotopes, Deuterium, Organics, Non-exchangeable h, Vapour equilibration, Drying, Reproducible and accurate determination of theδ^2^H of non-exchangeable hydrogen of complex environmental organic samples

## Abstract

Non-exchangeable hydrogen-isotope (*δ*^2^H_n_) measurements of complex organic samples are used in forensics to determine sample authenticity, traceability, and provenance. However, *δ*^2^H_n_ assays of organics are usually complicated by uncontrolled “exchangeable hydrogen” and residual moisture contamination; hence, *δ*^2^H_n_ assays are persistently incomparable amongst laboratories. We introduce a revised technical solution (UniPrep2) to control hydrogen-isotope exchange and for robust online sample drying and vapour equilibration. The UniPrep2 device is coupled to a high-temperature thermochemical elemental analyser and continuous-flow isotope-ratio mass spectrometer. This technical solution empowers isotope analysts to:

• Conduct reproducible controlled vapour equilibrations of complex organic samples and standards to determine the *δ*^2^H_n_ values by controlling hydrogen-isotope exchange.
• Conduct online vacuum-oven evacuation with extensive helium drying without exposure to air to reabsorb or exchange hydrogen with ambient water vapour.

The protocol describes the operation of the UniPrep2 device and the step-by-step procedures needed to obtain accurate and precise *δ*^2^H_n_ values for a wide range of organic sample types. Two analytical approaches are described in detail; the Dual-Vapour Equilibration (DVE) approach, intended for determining *δ*^2^H_n_ for a complex organic environmental sample where matrix equivalent H isotope reference materials are not available, and the Comparative Equilibration (CE) approach, which is intended for routine high-throughput analyses of complex organic samples where at least two matrix-equivalent organic isotope reference materials with consensus *δ*^2^H_n_ values are being used. These standard operating procedures are envisioned to be a sound basis for advancing hydrogen-isotope analysis for different organic environmental matrices and studies.

## Graphical abstract

**Figure:**
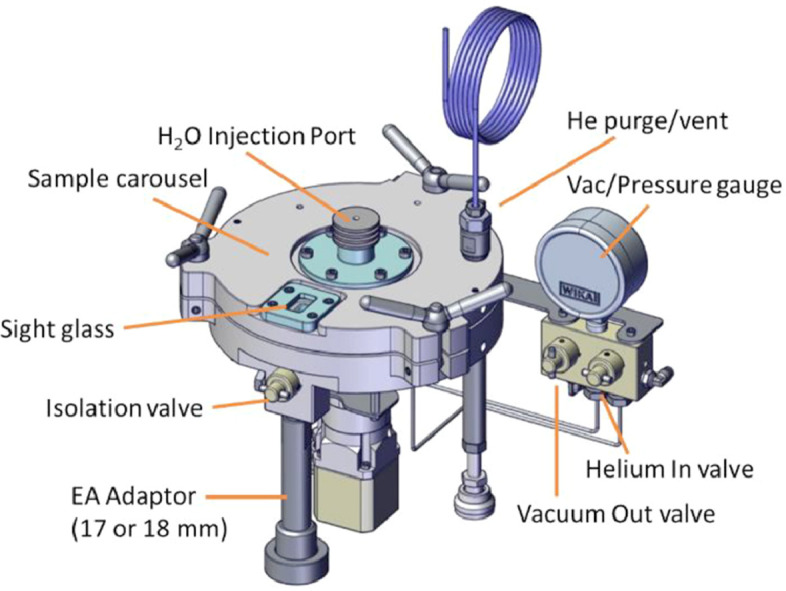

Specifications table

**Table:** 

Subject area:	Environmental Science
More specific subject area:	*δ^2^H Isotope Analyses of Organic Samples*
Name of your method:	Reproducible and accurate determination of the δ^2^H of non-exchangeable hydrogen of complex environmental organic samples
Name and reference of original method:	*Direct Submission*
Resource availability:	*Example dataset provided in Supplemental Materials*

**See Step 10**

## Introduction

Hydrogen stable isotope (*δ*^2^H) measurements of complex organic samples (e.g., hair, muscle, food, feathers, chitin, wood, biota, bone, etc.) are increasingly and broadly used in environmental forensics for the determination of sample authenticity and traceability in archaeology, in geology, and in ecological studies of animal migration and resource use [1], [2], [3], [4], [5], [6], [7], [8], [9]. These diverse hydrogen-isotope applications take advantage of the fact that non-exchangeable carbon-bound (C—H) hydrogen (*δ*^2^H_n_) in organic matter is derived from local environmental water and correlates with geographical provenance via predictable hydroclimatic hydrogen-isotope patterns in continental precipitation [10,11]. However, stable hydrogen-isotope assays of complex organic substrates are challenging because of variable fractions of so-called “exchangeable hydrogen” (e.g., O—H, N—H, S-H groups or not bound to a C), which can rapidly and uncontrollably exchange their hydrogen atoms with ambient (and spatially and temporally isotopically variable) H_2_O vapour in laboratory air during sample handling [12], [13], [14]. Moreover, organic samples can be weakly or strongly hygroscopic and adsorb contaminating ambient air moisture in the absence of or after well-controlled drying and handling [15]. It cannot be underestimated how difficult it is to thoroughly remove adsorbed residual moisture from environmental samples, which is essentially hydrogen-isotope (and ^18^O) contamination. Traditional offline sample drying methods to remove residual moisture (air, desiccator drying, vacuum oven, and freeze-drying) are time-sensitive operational techniques (e.g., dry @ 110 °C for 24 h) that offer no subsequent quantitative means to prove (isotopically) that all residual moisture has been removed. Laboratory air can be humid (volume fractions of H_2_O of 6000–16,000 ppmV). This is especially concerning when offline dried samples are re-exposed to air for some time during their handling and weighing into capsules. Accordingly, measurements of the *δ*^2^H isotope composition of complex organic environmental samples have remained persistently incomparable amongst worldwide laboratories owing to i) inconsistent approaches in controlling and eliminating hydrogen-isotope exchange, ii) variable or poorly reproducible approaches to sample drying that do not quantify elimination of trace residual moisture contamination, and iii) furthermore the unfortunate lack of a wide range of types of complex organic reference materials having well-known *δ*^2^H_n_ values. These three factors combined have seriously undermined the comparability, precision, and accuracy of organic *δ*^2^H data produced amongst stable isotope laboratories worldwide, as evidenced by poor outcomes in organic hydrogen-isotope proficiency tests [16,17]. Obtaining reproducible organic *δ*^2^H_n_ results amongst laboratories is crucial to the success of hydrogen-isotope provenance, authenticity, and environmental research studies and the development and interoperability of global environmental hydrogen-isotope databases [18].

**Fig. 1:**
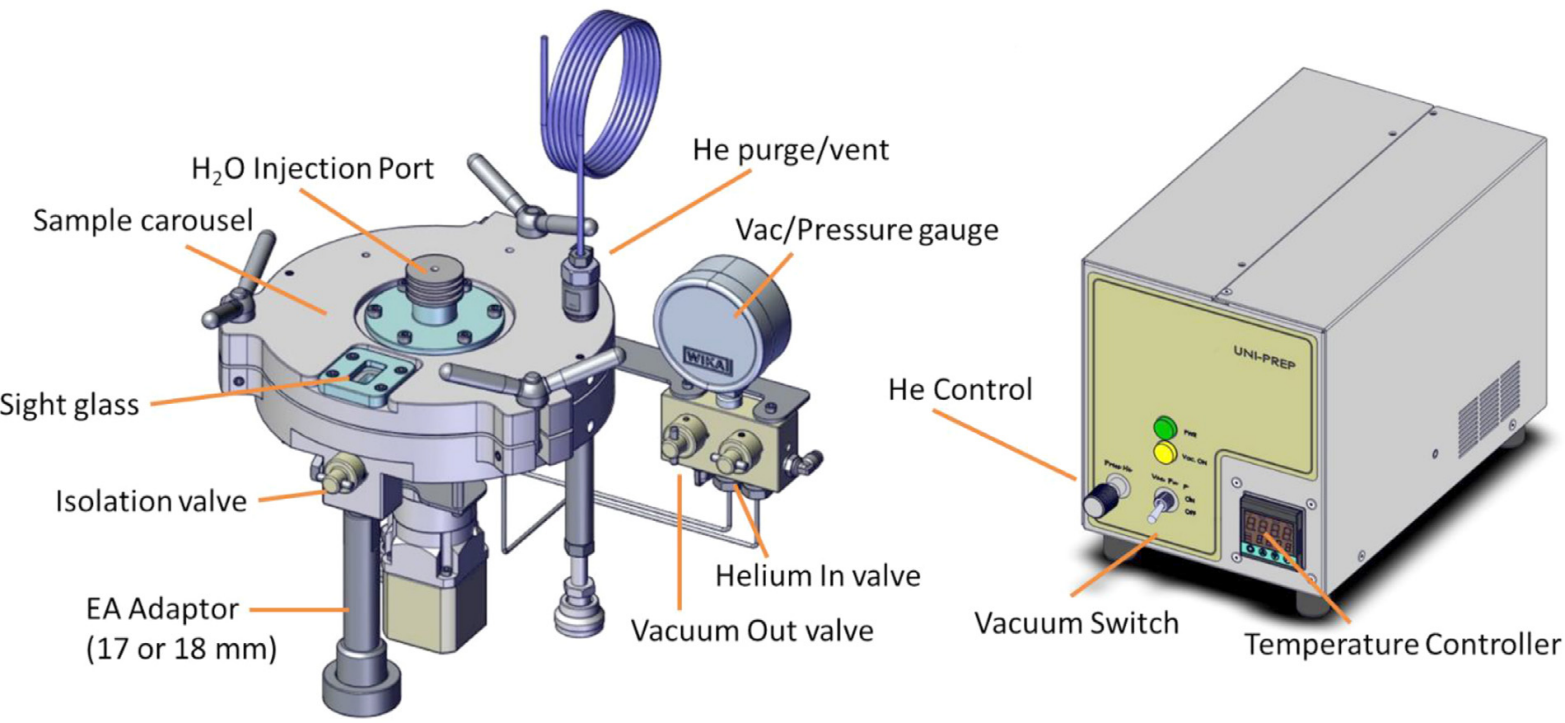
New UniPrep2 device and its control unit (not to scale).

In 2015, we introduced a technical solution to address the first two points, uncontrolled hydrogen-isotope exchange and online sample drying, by offering the Universal Preparation device (UniPrep™ - www.eurovector.it) [19]. The device (Fig. 1) is an online sample preparative device directly coupled to a high-temperature thermochemical (HTC; 1030–1450 °C) elemental analyser (EA) and a continuous flow isotope-ratio mass spectrometer (CF-IRMS). This technical solution empowered users to reproducibly conduct controlled vapour equilibrations of complex organic samples and standards to determine *δ*^2^H_n_ values and control hydrogen-isotope exchange, followed by online vacuum-oven drying with extensive helium flushing and drying [14]. Extensive online drying of analysis-ready samples with dry helium flushing ensures that samples are never re-exposed to laboratory air and uncontrollably reabsorb or exchange hydrogen with ambient water vapour, for example, during sample weighing or storage. The UniPrep2 offers a wide range of vacuum heating and helium drying options (from ambient T, 40–90 °C) to accommodate hygroscopic organic sample types. Moreover, the device can be used for online drying and zero-blank EA operations for *δ*^13^C and *δ*^15^N determinations of organic and inorganic samples. In short, the UniPrep device enables a vapour equilibration and online vacuum drying schema to control hydrogen-isotope exchange tightly and eliminate residual moisture contamination.

This paper aims to outline recent technical improvements to the original UniPrep and offer step-by-step procedures and tips to allow analysts to produce accurate and reproducible *δ*^2^H_n_ values for the most common analytical situations encountered in stable isotope laboratories. We also highlight deficiencies in scientific knowledge related to various organic samples regarding operational and analytical practices with the UniPrep2 that remain to be further explored by the research community. This paper assumes that a UniPrep2 device has been installed and functioning on an EA and CF-IRMS system per manufacturer specifications.

## Two approaches: dual-vapour equilibration and comparative equilibration

**Fig. 2:**
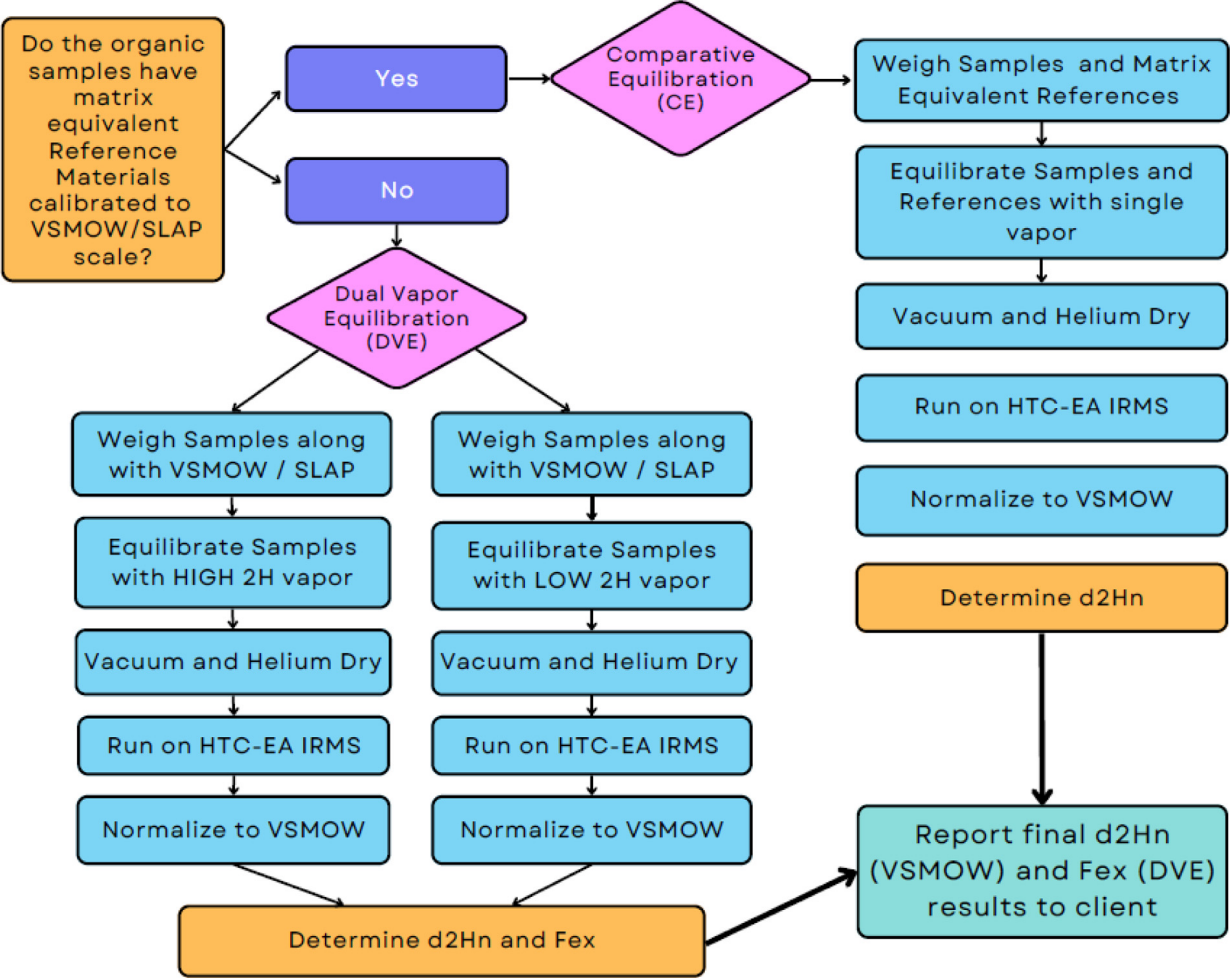
Decision flowchart for determination of δ^2^H_n_ of organic samples on the UniPrep2 using the DVE or CE approaches. The subscript n refers to non-exchangeable H.

The two general approaches used by the UniPrep2 to control hydrogen-isotope exchange in organic samples are known as *Dual-vapour Equilibration* (DVE) and *Comparative Equilibration* (CE) (Fig. 2). The Dual-vapour Equilibration (DVE) approach is intended for determining the *δ*^2^H_n_ values of complex organic environmental samples where matrix equivalent H-isotope reference materials are not (yet) available and hence require considerable extra analytical effort. By contrast, the Comparative Equilibration (CE) approach is far more straightforward and intended for routine and high-throughput analyses of complex organic samples (e.g., keratins, wood) where at least two isotopically separated matrix-equivalent H isotope reference materials with consensus *δ*^2^H_n_ values are readily available.

The most encountered scenario is the need for *δ*^2^H_n_ analyses for various classes of complex organic samples for which matrix-equivalent *δ*^2^H_n_ isotopic reference materials with consensus values have not been developed or are unavailable. Recently, a list of hydrogen-isotope reference materials with well-established consensus *δ*^2^H values has been made available [20], [21], [22]. These organic sample matrices will require the DVE approach [[12], [13], [14],^23^] (i) to quantify the sample's fraction of exchangeable hydrogen (*ƒ_ex_*) and H mass fraction and (ii) to subsequently derive its *δ*^2^H_n_. The DVE approach requires two separate analyses of the same sample, each controllably exposed to a sufficiently isotopically distinctive water vapour, after which samples are extensively dried and analysed, preferably online. The extent of *δ*^2^H isotopic response of each sample exposed to different vapours is solely a function of the fraction of its exchangeable hydrogen (assuming no residual moisture), which can be used to deduce the non-exchangeable hydrogen fraction that is of interest. For example, an organic sample with no exchangeable hydrogen (e.g., olive oil, polyethylene) should exhibit no *δ*^2^H response to different isotopic vapour exposures after drying [24]. In contrast, standard materials having considerable exchangeable hydrogen (e.g., cellulose) will give a more substantial response [25]. The DVE approach can be applied to all sorts of complex organics, for example, algae, particulate or soil organic matter, honey, fruits, leaves, and other complex organics for which *δ*^2^H_n_ reference materials with well-known consensus values have not been developed. The DVE approach requires concurrent analyses of water references having known *δ*^2^H values to determine the *δ*^2^H_n_ of the samples and ensure the results are anchored to the VSMOW-SLAP primary reference scale (see Step 10 below).

The second, more widely-used scenario is the CE approach [24], where two or more isotopically widely separated and organic reference materials having well-known consensus *δ*^2^H_n_ values on the VSMOW-SLAP scale are used for data normalization and which are reliably quantified for their *ƒ_ex_* values. Under the CE approach, unknown organic samples, their exchangeable hydrogen properties, and hygroscopicity are presumed to have been pre-established by DVE and to be (sufficiently) identical to the consensus hydrogen-isotope reference materials (e.g., use hair hydrogen-isotope references with unknown hair samples). In this case, a single vapour equilibration and online drying are equally applied to all samples and reference materials in a single UniPrep2 analysis batch. The CE approach has proven to be highly reproducible and robust amongst laboratories, is fast and less laborious than other methods, and hence is a preferable approach, provided that appropriate matrix equivalent reference materials exist [14,21,22,26]. Unfortunately, few organic *δ*^2^H_n_ reference materials currently exist having certified values [14,21,22,26], and some of these have been revised several times over the past decade as hydrogen-isotope analytical methods evolved [27]. A few current CE-ready *δ*^2^H_n_ reference materials are wood powders (USGS54a, USGS55a, USGS56a) and keratin powders (CBS, KHS) available from https://www.usgs.gov/labs/reston-stable-isotope-laboratory/reference-materials-and-calibration-services. Other new complex organic reference materials intended for eventual CE use for the determination of *δ*^2^H_n_ measurements are under development, and hopefully for a broader suite of organic sample matrices. A decision flowchart for deciding whether the DVE vs CE approach should be used with the UniPrep2 device is shown in Fig. 2.

It should be acknowledged that unacceptable approaches for organic *δ*^2^H are routinely published. These approaches include ignoring the hydrogen-isotope exchange issue, reporting organic sample *δ*^2^H values against inappropriate (e.g., inorganic mineral) isotope reference materials [28], or using the CE approach for samples using *δ*^2^H_n_ reference materials not matrix equivalent, thereby guaranteeing that results will be incomparable with other laboratories, as seen for example in [29].

## Modifications to the UniPrep

Since the original UniPrep was launched in 2015 and was incorporated by stable isotope laboratories, feedback from analysts recommended some technical improvements to the UniPrep device as well documentation of optimal and reproducible DVE and CE procedures for the device in connection with HTC EA and the CF-IRMS systems for the determination of *δ*^2^H_n_ values in complex organic samples. The UniPrep2 device is compatible with CF-IRMS systems from ThermoFisher (e.g., Flash HT Plus, EA-IsoLink, TC/EA), Eurovector (EA3000, Satellite), Hekatek (HTC), and with various interface systems (e.g., Conflo IV) that connect EAs to the UniPrep2 device using a serial connection and automated carousel software trigger. Here we document analytical procedures for the latest UniPrep2 using a ThermoFisher Flash HT EA system connected to a ThermoFisher DeltaV CF-IRMS. It should be noted that our examples are transferable to other HTC EA and CF-IRMS systems with minor technical or software modifications.

Compared to the original UniPrep device, the new 2022 version (Fig. 1) incorporates necessary modifications that improve the device's performance, reliability, and throughput. These improvements include:

• Elimination of some identified cold spots (where vapour could condense) at the bottom of the carousel by removing the motor housing from the base and fully incorporating the EA isolation valve into the heated zone.
• A helium-pressurized triple-O-ring motor axel housing to eliminate potential vapour or helium losses from the device during its use (e.g., no leakage).
• Addition of a helium purge valve with a capillary outlet that allows users to vent or flush the carousel for sample drying (or as zero-blank) with controlled carousel helium flushing rates from 85 mL/ min (@ 0.8 bar helium pressure) to 150 mL/ min (@ 2 bar helium pressure).
• The carousel tray size was increased to 80 (79 usable) samples (from 50 (49 usable)) and further optimized for 3.5 mm O.D. by 3–9 mm silver capsules for  *δ*^2^H and *δ*^18^O measurements and all crushed tin capsules for *δ*^13^C and *δ*^15^N measurements.
• An improved vacuum system rapidly evacuates the carousel via capillary to <5 mbar pressure (for vacuum vapour equilibration) and for evacuating air and residual moisture.
• A viewing glass reveals the internal user-adjustable index counter and a direct view into the EA-HTC reactor to check for sample blockage.
• Compact cartridge heaters, a thermocouple, and a reduction in cabling (not depicted).
• A helium control valve allows matching carousel helium pressure to the EA system to minimize pressure pulses and carrier flow variations to the CF-IRMS when opening the isolation valve.
• Incorporating a vacuum pump, temperature controls, and a helium pressure regulator into a single control box.
• Addition of EA connection fittings for 17- or 18-mm O.D. HTC ceramic or quartz reactor tube systems.
• Precise isothermal heating and temperature control of the carousel for drying and vapour equilibrations ranging from 40 to 90 ± 0.1 °C or may be used at ambient temperature.

### How it works

Regarding Fig. 1, the UniPrep2 operations are as follows (elaboration of technical details specific to *δ*^2^H_n_ measurements of organics are discussed in later sections):

• The “Isolation valve” opens/closes the UniPrep2 to the analytical circuit of the EA and the CF-IRMS. When closed, helium carrier gas from the EA flows normally through the EA into the CF-IRMS (e.g., EA background).
• Samples and references are loaded in 3.5 mm O.D. x 3–9-mm silver capsules are placed into the 79-position sample tray (Fig. 4).
• An adjustable indexing disc (Fig. 4) shows the current sample being analysed through the sight glass and can be “zeroed” without rotating the entire carousel.
• The “Vacuum Out” valve on the manifold opens/closes the vacuum pump line to the autosampler. When the cover is closed (and the “Isolation valve” is closed), the vacuum pump evacuates air and moisture from the carousel and samples to < 5 mbar pressure.
• A Pressure gauge on the manifold monitors the chamber pressure (e.g., helium flushing) or vacuum status (e.g., for vacuum drying).
• The “H_2_O injection septum port” allows users to inject liquid water (up to 150 µL of H_2_O) into the evacuated chamber for isothermal timed static sample-vapour isotopic equilibrations.
• The “Helium In” valve (with the “Isolation valve” closed) allows helium to fill or flush the sample chamber and is pressure adjustable to match the Elemental analyser pressure before opening the “Isolation valve” to the CF-IRMS.
• The “He purge” valve allows for a constant flush of helium through the sample carousel up to approximately 150 mL/min with the “Isolation valve” closed or allows for venting to open the carousel.
• The “Temperature Controller” sets the precise carousel temperature (40–90 ± 0.1 °C) for vapour isotopic equilibrations, outgassing, and online vacuum drying. Optionally, the device can be operated at ambient temperature.

### Analysing samples for organic δ^2^H_n_ values

*Step 1. Ready the CF-IRMS*

The CF-IRMS system should be readied for *δ* ^2^H stable isotopic analyses per manufacturer specifications, including ensuring the best IRMS focus tuning for low and reproducible H_3_^+^ contribution, sensitivity, and stability testing in a continuous-flow mode. Depending on the dynamic range of the IRMS system, the organic samples and reference sample masses should be targeted to achieve roughly the same H_2_ signal, typically between 6000 and 8000 mV (instrument specific). An ion source linearity test should be conducted by measuring an organic standard by ranging its mass, typically between 200 and 1000 µg, to determine the response of the CF-IRMS to varying H_2_ yields from sample pyrolysis. In most cases, an ion source linearity correction can be derived using offline data processing software like *LIMS for Light Isotopes* [30] to correct the results for sample mass/H_2_ yield-dependant isotopic variance.

*Step 2. Prepare the HTC-EA reactor tube*

The HTC pyrolysis reactor configuration will partly depend on the make of EA being used, the reactor tube length, EA fittings, EA reactor temperature range available, and whether *δ*^2^H_n_ and *δ*^18^O measurements for organics are required. Most EA systems have reactor tube connection fittings for either 17- or 18-mm O.D. ceramic or quartz tubes with lengths ranging between 450 and 470 mm. The correct UniPrep2 EA adaptor should be installed for the selected reactor tube.

**Fig. 3:**
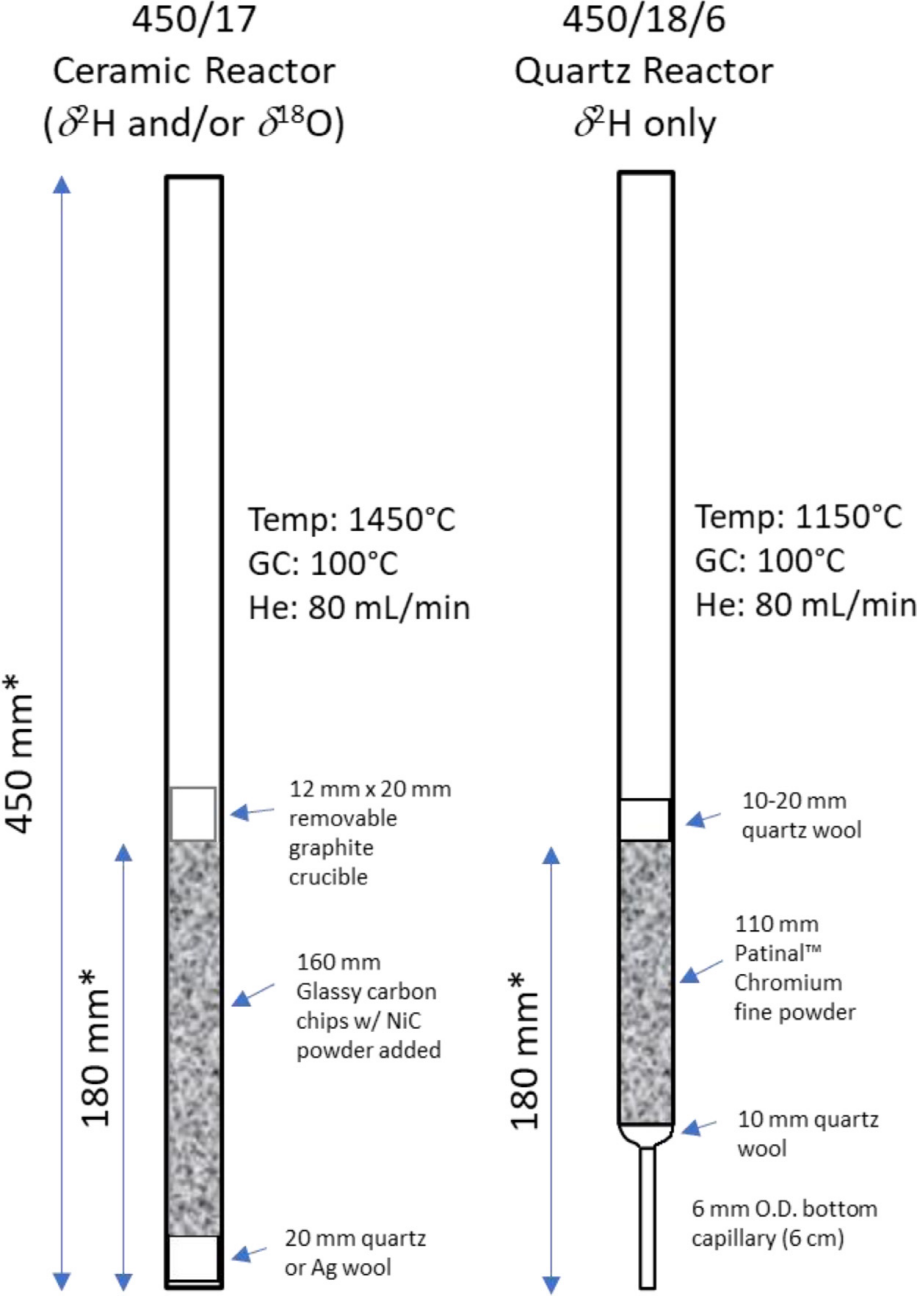
Recommended HTC reactor configurations for use with the UniPrep2 for δ^2^H_n_ or δ^18^O analyses of organic samples. The capillary quartz chromium reactor is recommended for nitrogenous or halogenic complex organic samples. The loading dimensions of the ceramic or quartz reactor tubes depend on the EA system used and the connection fittings. The configuration depicted is for ThermoFisher Flash HT / TC/EA and EA Isolink systems. The quartz capillary reactor requires an optional 6 mm bottom connection fitting. The UniPrep2 EA interface has top connection adaptors for 17-or 18-mm-O.D. Quartz or ceramic reactor tubes.

Fig. 3 illustrates two recommended reactor tube configurations compatible with ThermoFisher Flash HT, TC/EA and EA-Isolink systems. The chromium-filled reactor (Fig. 3, right) is particularly recommended for *δ*^2^H_n_ measurements of complex organic samples that contain nitrogen (e.g., proteins) or halogens because the chromium metal catalyzes the removal of CO and nitrogen and reduces bias by potential hydrogen-isotope fractionation due to formation of non-target by-products (e.g., HCN, HF, HCl) [31], [32], [33]. Moreover, carbon and (most) nitrogen are scrubbed by chromium to produce clean H_2_ peaks without any CO or CH_x_ peaks and with minimal N_2_ peaks for nitrogenous samples.

The 18-mm-O.D. Quartz capillary chromium reactor (Fig. 3, right) is filled with high-purity Patinal™ fine chromium metal powder (<0.3 mm) up to the hot zone and is under and overlain by quartz wool plugs. This configuration is workable for approximately 2000 samples before the chromium reagent is used up. The uppermost 2-cm quartz wool (formed as a dimpled “nest”), or a graphite crucible, collects the silver from the sample capsules and allows for easy reactor cleanup. Cleanup involves cooling and removing the quartz reactor from the EA, rolling the solid silver nugget out, and re-installing the tube. Cleanout will be needed after approximately 8–10 gs of silver has accumulated from the sample capsules and to prevent reactor blockage (NB: silver capsules range in mass from 14 mg (thin-walled) up to 120 mg (thick-walled) each). Notably, and as a precaution, be aware that molten silver in direct contact with chromium reagent will bond with the chromium and quartz tube to make cleanout and tube reuse almost impossible, thus one should ensure there is 1–2 cm of quartz wool separation between the silver capsule landing zone and the chromium. The quartz capillary reactor tube setup is beneficial for other reasons: it can be run at a lower temperature (max 1150 ºC), the bottom capillary produces sharper H_2_ chromatography due to lower gas dispersion in the fine chromium powder, 40 % less chromium reagent is used compared to a straight quartz tube, and quartz is transparent for convenient viewing during reagent filling. Moreover, HTC EA oven life is extended by avoiding operation at highly elevated temperatures.

One disadvantage of quartz tubes is that dual-isotope (*δ*^2^H and *δ* ^18^O) analyses are impossible due to carbon removal by the elemental chromium reagent and lower temperature. Our testing showed that chromium reactor temperatures below 1150 ºC are not recommended, as revealed by CH_x_ and CO peaks increasingly rising at lower temperatures on the Thermal Conductivity Detector (TCD), primarily when chromium reactors are run much lower than 1100 ºC. The EA GC oven should be hot (100 °C) and fitted with a 1.5-m packed “oxygen separation” column to ensure a distinct H_2_ gas peak. Helium flow rates through the reactor should not exceed approximately 80–90 mL/min to allow complete sample conversion to H_2_ gas. The EA-TCD should be calibrated and monitored to quantify sample hydrogen yields. Sample run time will be approx. 300–400 s and is EA-specific.

The 17-mm-O.D. ceramic reactor (Fig. 3, left) is the more common configuration in stable isotope laboratories. The ceramic reactor tube is filled with inert glassy carbon chips above an approximately 2 cm silver or quartz wool bottom plug up to the hot zone (a glassy carbon insert tube is optional). For effective pyrolysis, it is recommended to disperse a few grams of NiC powder amongst the upper glassy carbon chips because effective pyrolysis to H_2_ and CO gas requires readily available active carbon. Optionally, instead of adding NiC powder, initially running 4–5 carbon-rich samples (e.g., NBS 22 oil) to pre-condition a new C reactor will supply sufficient available carbon to form H_2_ and CO for later samples. The EA GC oven should be hot (100 °C) with a 1.5 m oxygen separation column to ensure clean separation of the eluting H_2_, N_2,_ and CO gas peaks. Helium flow rates through the carbon reactor should not exceed 80–90 mL/min to ensure complete conversion to H_2_, N_2,_ and CO gas [34], which can be readily monitored if the EA is fitted with a TCD. These ceramic tube carbon-filled reactors are typically run at 1350–1450 °C for organic *δ*^2^H analyses. Some EA configurations offer a reverse helium flow configuration and bottom feed adaptor, which is not needed for *δ*^2^H (and reduces sample capacity). A 12.7-mm-O.D. x 2.4-cm thin-walled graphite crucible (Alpha Resources P/N: AR053HD) placed on top of the glassy carbon chips will catch the silver capsules for easy cleanup after 8–10 gs of molten silver has accumulated. After cooling and removing the reactor, the silver “nugget” rolls out of the crucible. The ceramic reactor can be reused for at least 2000–3000 samples before replacing the inert glassy carbon chips. Sample run time will be 300–500 s and is EA and CF-IRMS specific. After each new reactor installation, an automated or manual EA system helium leak check is strongly recommended.

*Step 3. Preparing the Organic Samples and References*

For the determination of *δ*^2^H_n_, values, the organic samples and references are weighed into silver capsules. They are isothermally and statically equilibrated to either one (CE Method) or two (DVE Method) isotopically known water vapours with sufficient time for full hydrogen-isotope exchange without any hindrances caused by crushing or tightly closing the sample capsules. Silver capsules should be selected for the smallest size adequate to hold the samples and references without overfilling and allow total vapour exposure. Round-bottom “liquid” silver capsules 3.5 mm O.D. x 4–9-mm tall that weigh 70–120 mg each are ideal, compared to thin-walled 14-mg thin-walled EA silver capsules. Organic samples and references are typically weighed out between 200 and 700 μg (depending on the total sample mass to achieve a similar hydrogen mass) into the silver capsules. The masses of the samples and standards should be carefully and accurately H_2_-yield-matched (based on total hydrogen concentration) to obtain approximately the same EA and CF-IRMS H_2_ peak height and area to avoid significant ion source linearity deviations. Pre-testing of unknown samples to estimate the hydrogen content may be required. In many cases, biological organic samples or foods may contain significant fractions of ^2^H-depleted lipids or volatile organic compounds (VOC), which should be removed by using a preparative solvent (e.g., 50:50 v/v methanol-choloroform solution) removal step, because lipids will especially masque the non-exchangeable hydrogen signal [35].

One longstanding challenge for *δ*^2^H_n_ measurements and vapour equilibration of complex organic samples was how to ensure and control the sample vapour exposure and later facilitate the complete removal of equilibration vapour and residual moisture for both samples and reference materials. Traditionally, EA samples are tightly encapsulated or crushed into thin-walled silver capsules; hence, hydrogen-isotope exchange and residual moisture removal are exceedingly challenging to guarantee and may even be prevented by blockage and sealing. Previous adaptations that enable vapour exposure for equilibration and drying included loose encapsulation or micro-puncturing of the silver capsules to facilitate vapour and gas exchange [36]. Major drawbacks to these operational approaches include spillage, capsule rupturing, user skill, uncontrolled blockage, and a general difficulty in small sample handling with thin-walled silver capsules. Once samples and standards are encapsulated, they are often stored in the laboratory before hydrogen isotope analysis. They can experience differential times and rates of ambient H_2_O exposure, whether tightly or loosely encapsulated, stored on a shelf, or placed in a desiccator.

To overcome uncontrolled vapour blockage during equilibration, the UniPrep2 carousel was redesigned to use the robust 3.5-mm-O.D. by 4–9-mm tall round-bottomed ("liquid") high-purity silver capsules with the capsules left fully open (no crushing or sealing required). Allowing the silver capsules to remain open-topped inside the carousel ensures that all samples and references are equally and fully exposed to the vapour for hydrogen-isotope equilibration, vacuum drying, and helium drying without any hindrance to isotope exchange or vapour movement. These solid silver capsules cannot tip over and lodge sideways in the carousel and so have a minimal risk of spilling their contents inside the UniPrep2. Moreover, a gentle carousel rotation drops capsules into the HTC reactor without tipping. Evacuation and helium flushing are made through small capillary tubes to avoid abrupt carousel pressure changes (e.g., to eliminate powders exploding out of an open capsule during evacuation).

For the DVE approach, at least two water references with known consensus *δ*^2^H values are required because the *δ*^2^H_n_ values must be anchored to the VSMOW-SLAP water scale. However, using water for EA standards is almost impossible to dispense and cold-weld into small silver capsules without compromise, and so to be later unaffected by heat or vacuum on the UniPrep2 device. Instead, we strongly recommend using USGS reference waters supplied pre-sealed in silver microtubes. These silver microtubes are unaffected by heating or vacuum in the UniPrep2 carousel and yield highly reproducible results. Notably, we suggest using VSMOW and SLAP or the appropriate *δ*-value range of pre-calibrated waters provided in 0.15 mL or 0.25 mL silver microtubes by the U.S. Geological Survey (https://www.usgs.gov/media/files/rsil-report-stable-isotopic-composition-reference-material-vsmow-025). The *δ* range of reference waters selected for DVE should fully bracket anticipated *δ*^2^H_n_ range of the organic samples *after* they are exposed to the equilibration H_2_O vapour (see Equilibration), especially if using highly ^2^H-enriched or depleted equilibration vapours. For a control standard, one can use water reference materials in silver microtubes or other isotopically proven organic materials – but importantly, the control should not have exchangeable hydrogen. Recommendations for reasonable non-exchangeable controls include NBS22a oil (available in 0.15 or 0.25 mL in silver tubes) or USGS 77, a polyethylene powder that can be weighed into the open silver capsules like the unknown samples. USGS77 is especially useful because it should not respond to any vapour equilibration; hence, isotopic deviations of this control standard from the expected value would show that the carousel may not have been sufficiently dried or helium flushed. The only reference materials required for the CE approach are two matrix equivalent complex organics and USGS77 as a control to correct for instrumental drift and linearity.

Ideally, unknown organic samples for *δ*^2^H_n_ measurement are pulverized (to maximize equilibration vapour exposure and ensure isotopic homogeneity), but this is entirely dependant on the scientific purpose and the types of sample materials (see the review in [35]). For example, keratins (e.g., feathers, hair) are extremely difficult to pulverize, are often highly electrostatic in powder form, and so may be analysed as small cuttings for handling convenience [15,37]. Other samples, such as honey, are semi-solid at room temperature and cannot be powdered. Informed scientific discretion is advised about sample pulverization and the subsequent handling. Accurate weighing of small dry organic samples is challenging and often prone to static charge and room drafts leading to inaccurate masses; hence, using anti-static mats, using ionizing guns, and wearing light cotton clothing will minimize these annoyances.

For weighing an organic sample, an internally round-bottomed silver capsule (the external base is flat and stands upright) is tared using a high-precision (4–5 decimal) analytical microbalance. The samples are weighed using a clean stainless-steel spatula or cut with a blade to within ± 50 µg of the target weight. The target weight of the organic sample must be pre-determined using its estimated or pre-measured total hydrogen mass concentration, the CF-IRMS peak area of the sample, and the reference materials being used. For example, when 0.25 mL (250 µg) of USGS reference waters in silver microtubes are used (H_2_O is 11.1 wt. % hydrogen by mass), a typical H_2_ peak area on the CF-IRMS is 100 vS (8000 mV peak height); hence, the unknown organic samples should have a target weight that produces approximately the same H_2_ peak area and size. For keratins and wood samples (6–7 % hydrogen by mass), the comparative sample weight needed is around 400 µg, and for USGS77 polyethylene powder (14.5 % hydrogen by mass) is around 200 ug. The recorded sample in the silver capsule is carefully transferred from the microbalance into a 96-well position-numbered plastic plate. This plate with samples and references should be secured with a protective plastic cover for storage until hydrogen-isotope analyses. When storing “open-top” silver sample capsules for later analysis, care must be taken to avoid shaking or tilting the plastic storage plate to avoid capsule tipping and sample spillage inside the tray. Moreover, silver capsules quickly oxidize under moist light conditions; hence, they are best stored under dry and dark conditions or analysed immediately after sample preparation.

For the DVE approach, splitting a single UniPrep2 carousel loading into two distinctive runs is preferable. The first half of the carousel is loaded with samples and references for the initial low *δ*^2^H vapour-equilibration sequence, and the second half of the carousel is loaded with replicates of the samples and references for the high *δ*^2^H vapour-equilibration sequence (see the examples in the Excel file in Supplemental Materials). After the first vapour-equilibration sequence, the run is stopped, the UniPrep2 “Isolation valve” is closed, the carousel is evacuated, and followed by injection of the high *δ*^2^H vapour for equilibration. The advantage of using the split template is that the DVE process is completed in a single batch with no changes to the CF-IRMS tuning settings or to the chromium or carbon reactor. An alternative approach is to analyse a full carousel with a low *δ*^2^H vapour equilibration, clean out the reactor as required, and then repeat the entire carousel with a high *δ*^2^H vapour equilibration. Either approach is acceptable.

When ready for hydrogen-isotope analyses, the samples and references are transferred from the plastic storage plate into the UniPrep2 carousel wells and set up an appropriate CF-IRMS instrumental job list. An EA template comprises samples, references, and controls for the DVE and CE approaches. Example DVE and CE analysis templates for the UniPrep2 are found in Supplemental materials.

**Fig. 4:**
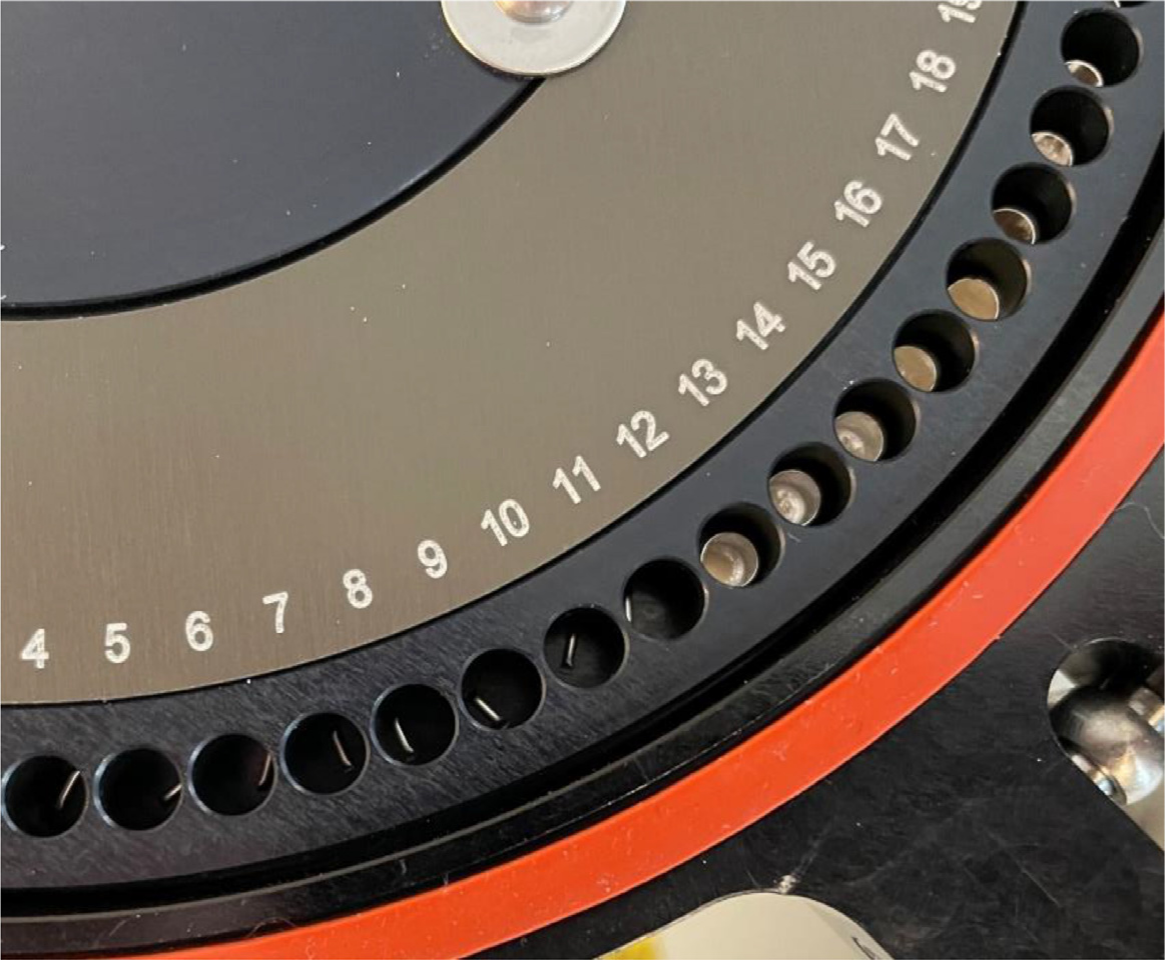
UniPrep2 carousel loaded with isotopic reference waters in silver microtubes (USGS47, USGS53 in pos 4–11) and the unknown organic samples in open-topped silver round-bottom capsules (pos 12–19). The adjustable sample counting ring is shown along with the red sealing gasket.

The UniPrep2 carousel “zero” position should be manually aligned and verified to be slightly past the centre by approximately 1–2 mm, which ensures that open-top silver capsules do not lodge sideways in the EA opening. Manual alignments to the carousel must be made with the UniPrep2 power turned off to avoid damaging the electronic stepper motor. Before loading the samples, loosen the inner counter ring holding screw (Fig. 4) and rotate the counting ring, so that position “0” (to “Isolation valve” opening) lines up with the EA starting position, and then retighten the screw. Samples are placed into wells 1–79, not into the Isolation valve “0” position, as with some EA autosamplers.

Step 4. Setting UniPrep2 Equilibration and Drying Temperature

Establishing a proper temperature for online static vapour equilibration and later drying on the UniPrep2 is essential for analysing each type of organic sample matrix. Generally, while hotter would be better (>40 to 90 °C) for achieving faster vapour equilibration, removal, and sample drying, the appropriate temperature will depend on the type of organic material being analysed for its *δ*^2^H_n_ value. Some organic matrices may not be resilient to higher temperatures if coupled with a hot vapour exposure (e.g., vacuum deterioration– see insert). In contrast, others, like hair, feathers, and chitin, are resilient to high temperatures >120 °C and vapour. Others, such as benzoic acid, may sublime, degrade, or change their structure with resulting hydrogen-isotope effects at lower temperatures. Regardless, the vapour equilibration step and the vacuum drying on the UniPrep2 should be conducted at the same temperature.

*Caution*: There are no established rules for which equilibration temperature to use. For some complex organic matrices, the effect of heating in combination with vapour (e.g. denaturation, sublimation) and the impact on the *δ*^2^H_n_ value remain unknown [19]. It is, therefore, incumbent on the researcher to understand their sample matrix and its potential susceptibility to structural or hydrogen-isotope alternation by applying heat, vapour, and vacuum weighed against the practicality and trade-offs in using higher heat to remove residual vapour. Pre-testing may be needed at various temperatures and visual or microscopic evaluation of the sample materials to decide on an appropriate heat vapour equilibration and drying regime. For temperature-resilient materials, we recommend using 60 °C as a starting point, and one can explore the effects (or lack of) by using higher or lower temperatures. Our testing has shown that some resilient organic materials like keratin and chitin (e.g., feathers, claws, hair) produce identical *δ*^2^H_n_ results between 25 and 110 °C treatments using CE. Also, bear in mind that the boiling point of water is significantly reduced at low vapour equilibration pressures (approximately <5–10 mbar in the carousel). Conversely, residual water is always challenging to remove from metal and other surfaces without applying heat, vacuum, and helium flushing (e.g., IRMS bakeouts). Ultimately, a sound regimen for an organic sample matrix can only be successful and considered robust if *δ*^2^H_n_ determinations can be fully reproduced in another laboratory adopting identical standard operating procedures.

After loading samples and reference silver microtubes into the carousel, close and tighten the carousel cover, ensure the “Isolation valve” and “He purge” valve is closed, and ensure a septum is in the “H_2_O injection port”. Turn on the UniPrep2 power switch (rear), if not already on, and then use the “Temperature Controller” on the control box to set it to the required vapour equilibration and drying temperature. Always use caution and wear heat-protective gloves when touching the hot surface of the UniPrep2 carousel. During the carousel heating, the samples can be evacuated to remove moisture and room air from the carousel. Turn on the membrane pump switch at the front of the control box and gently open the “Vacuum Out” valve on the UniPrep2 manifold. The gauge's pressure should drop and stabilize at <5 mbar. Continue evacuating the carousel and samples at the setpoint temperature and then continue to Step 5.

Step 5. Conducting Static Vapour Equilibration for Hydrogen-Isotope Exchange

All samples and references will need to be run twice for the DVE approach. Each sample set is statically equilibrated under heated vacuum conditions with two isotopically known reference waters having *δ*^2^H values that span the *δ*^2^H values of the samples. Ideally, the waters used for the static vapour equilibration are distilled or low in total dissolved solids to minimize salt buildup below the “H_2_O injection” port. However, minor salt residues can be wiped from the hydrophobic metal top inside the carousel. Generally, it is advisable to use two waters for vapour equilibration that are widely separated in their *δ*^2^H_VSMOW_ values by at least 300 ‰ and span the expected *δ*^2^H range of the unknown samples. Their *δ*^2^H values should be well-established relative to the VSMOW-SLAP scale. These injection waters must be carefully stored in sealable glass bottles just like reference waters to minimize isotope fractionation by evaporation over time [38]. Suppose a >300 ‰ range of equilibration water is unavailable. In that case, it is easy to make up a ^2^H enriched sample using laboratory-deionized water and gravimetrically add a precise amount of low-cost 99 % D_2_O to raise the *δ*^2^H value by >300 ‰. Establishing the *δ*^2^H_VSMOW_ value of these vapour injection waters is critically essential (see below), but water isotope analysis is beyond the scope of this paper.

For the CE method, the *δ*^2^H value of the equilibrating vapour does not matter (one may use laboratory-deionized water) because samples and references are all equilibrated with the same vapour and are normalized with the assigned *δ-*values of the matrix-equivalent reference materials, hence, use of distilled laboratory water for vapour CE equilibrations is convenient. Nevertheless, using water with a significantly higher or lower *δ-*value than the samples will allow for reliable detection of sample drying status, see below. Be aware that *ƒ_ex_* cannot be determined using the CE approach.

After the samples and references have been loaded and the carousel top closed and sealed by tightening clamps, verify that the UniPrep2 “Isolation valve” is closed. Switch on the UniPrep2 power (if not already on) and check that the “Temperature Controller” is at the correct equilibration temperature (40–90 °C). If using ambient room temperature for equilibration, set the “Temperature Controller” to 20–25 °C because the power must be on to activate the carousel (the controller defaults to 60 °C at start-up). Allow around 30–45 min for the temperature of the UniPrep2 carousel to stabilize. Ensure that the “He purge” valve on top of the carousel is closed, the “Helium In” valve is closed, and the 6.5 mm septum inside the injection port is sound. Turn on the “vacuum pump switch” in front of the control box and gently open the “Vacuum Out” valve to the carousel. Allow the carousel to be evacuated for approximately 30 min; the vacuum should be <5 mbar. After 30 min of pumping, close the “Vacuum Out” valve and observe the “Vac/pressure gauge” on the manifold – it should hold <5 mbar. If the pressure slowly or rapidly begins to rise, either the carousel top was not properly seated, the top “helium vent/in valve” is not fully closed, the “H_2_O injection port” septum is compromised, or the “Helium In” valve is not fully closed. (Caution: ensure that the “Vacuum Out” and “He in” valves are never open at the same time – this will evacuate the helium gas cylinder). If the vacuum holds after closing the “Vacuum Out” valve (it should contain the vacuum indefinitely), turn off the “vacuum pump switch” on the front of the control box. The control box membrane pump is intended only for carousel pumping only and should never be left running continuously.

Next, for DVE (low *δ*-value equilibration water) or CE, using a 100-µL glass syringe, obtain and then gently inject 30–100 µL (as required based on *ƒ_ex_*) of the low *δ*^2^H water (or DI water for CE) through the top septum injection port into the carousel. After the water injection, the pressure on the carousel gauge should slowly and visibly rise above 5 mbar, the degree of rise depending upon the carousel temperature and the amount of water injected. It will stabilize after approximately 3–5 min. A complete discussion of the amount of water needed for full vapour equilibration is found in [19]. It should be targeted to be 100-fold more than the estimated total exchangeable hydrogen of all the samples inside the carousel. Remember that the most amount of H_2_O that can be injected into the UniPrep2 under vacuum conditions is < 200 µL; otherwise, there is the risk of a liquid water phase condensing inside the carousel. Allow the samples to statically equilibrate with the injected water vapour at the set temperature for at least 1 h to allow ample time for the samples and references to reach hydrogen-isotope equilibrium [23]. Allowing longer vapour equilibration times (e.g., many hours) is acceptable, but bearing in mind the type of organic samples being analysed (e.g., thermal vapour degradation). At equilibration temperatures below 40 °C or ambient temperature, a longer static vapour equilibration time may be needed to reach hydrogen isotope equilibrium and should be figured out experimentally. For the DVE approach, this vapour equilibration procedure will need to be repeated with a duplicate set of weighed organic samples and references but by injecting the higher *δ*-value equilibration water the second time.

*Caution*: For many complex organics, the time needed to reach hydrogen-isotope equilibrium with vapour has to be experimentally determined, especially at ambient temperatures. Some complex organics may be inappropriate for heated vapour equilibration (e.g., organics that easily dissolve or sublime). Previous work has shown that 1–2 h vapour equilibration times are usually sufficient at elevated temperatures [23].

Step 6. Vacuum Pumping and Drying

Following the sample-vapour equilibration step, all H_2_O vapour inside the carousel must be removed. The samples are dried initially by evacuation and then by dynamic helium flushing to ensure complete drying. The vacuum pumping time depends on the samples' hygroscopicity and moisture retention capacity. As a starting point, the recommended vacuum time pumping is 30 min while monitoring the pressure gauge on the carousel manifold until it is steady at <5 mbar vacuum. Check to ensure the EA “Isolation valve” and “Helium In” valves are closed when vacuum drying. Turn on the “vacuum pump switch” in front of the control box. Gently open the “Vacuum Out” valve to ensure that powdered samples do not explode out of their capsules. Evacuate for 30 (or more) minutes. After evacuating, close the “Vacuum Out” valve. All the UniPrep2 manifold valves should be closed, and the carousel should hold at <5 mbar pressure. It is not recommended to forgo the H_2_O evacuation step and skip directly to helium flushing because the purge capillary inside diameter is exceedingly small and could become blocked with condensing water vapour.

Step 7. Helium flushing and sample drying

The helium flushing and drying step that follows the initial vapour removal by evacuation is the final and critical step to ensure that samples are thoroughly dried online. The helium pressure and the capillary length control the helium flushing rate exiting the “He purge” valve. Using the factory-supplied capillary, a control panel helium pressure of 0.8, 1.0, and 2.0 bar helium should give carousel flushing rates of approximately 80, 100, and 150 mL/min. As a starting point, is it recommended to use 100 mL/min (1 bar) to flush the carousel, mainly because this helium pressure is close to the operating pressure of the EA, thereby minimizing pressure changes to the EA and CF-IRMS when opening the “Isolation valve.” If higher or lower helium flushing rates are needed, increase or decrease the helium pressure with the needle valve (max is 2 bar). Do not forget to set the pressure back to the EA helium operating pressure when the drying is completed. If a higher helium flow than 150 mL/min is desired (and not exceeding 2 bar helium), the vent capillary can be shortened by a few cm and retested for a faster flow rate.

Set the helium pressure on the control box to 1 bar, gently open the “Helium In” valve on the manifold and allow the carousel pressure to stabilize. Next, open the “Helium purge” valve on the top of the carousel by rotating it counterclockwise by one complete turn (note: two turns will vent the carousel). This 1-turn setting allows pressurized helium to enter the carousel to pass over the samples and flow through the vent capillary at the set flow rate. If a digital flowmeter is available, check the helium flow rate at the end of the purge capillary and adjust using the helium needle valve on the UniPrep2 control box to get the desired flow rate. Carefully observe the outlet tip of the purge capillary for water droplets appearing after the first few minutes. With the initiation of the helium flushing, a few droplets may appear as the helium carrier gas effectively removes the remaining adhered water from the inside surfaces of the carousel and the samples.

The time required for helium flushing can vary to remove all residual moisture and will need to be experimentally determined based on the sample matrix type and the hygroscopicity of the samples. For example, some complex organic samples like keratins may require <2 h of helium flushing, and other types of organics may require 4 h, or longer (next section).

After the helium flushing and drying step is complete, check and readjust the helium pressure on the needle valve to match the EA and CF-IRMS system carrier pressure. Then tightly close the “He purge” valve (clockwise rotation). All valves on the UniPrep2 should be closed, and the carousel is now pressurized with helium. The samples are now ready for hydrogen-isotope analyses after a dryness check. As one final check before drying and H isotope analysis, using a handheld helium leak detector, check all of the UniPrep2 fittings, connections, and valves; the EA system should be helium leak-free.

(NB: given the recent helium supply crisis, the flush drying step could be done using dry N_2_ gas; however, this would require the user to add some gas fittings and valves to allow switching between N_2_ and He, bearing in mind that helium is the more effective vapour carrier).

Step 8. Sample Dryness Check

Before starting the hydrogen-isotope analyses, a sample dryness check using the CF-IRMS is required until a routine drying protocol is determined. After opening the UniPrep2 sample carousel to the EA reactor, any residual H_2_O emanating from the samples or the carousel and through the HTC reactor should produce a measurable rise in the IRMS H_2_ signal above the “EA background.” Unlike traditional offline drying methods, this approach provides a quantitative means to test for drying completeness because the IRMS is an ultra-sensitive detector of residual H_2_O carried through the HTC reactor. There are three IRMS background readings to consider:

• CF-IRMS background: IRMS is *closed* to the EA (or 100 % sample dilution on Conflo) – this is typically <20–40 mV H_2_ signal and should be at once stable.
• CF-IRMS background: IRMS *opened* to the EA-HTC reactor (no sample dilution on the Conflo) – this background is typically higher but <120–300 mV depending on the type of reactor, its operating temperature, and IRMS tuning. After opening the EA reactor to the CF-IRMS, it can take 30 min or longer for the system to stabilize and reach a stable “EA background.” The best approach is to set up a time scan (e.g., 2 h) and monitor the H_2_ signal over time. Typically, a low and stable EA background is achieved in <15–30 min.
• The UniPrep2 background or dryness H_2_ signal (see below): Carousel opened to EA and CF-IRMS.

To assess the dryness of the samples, the UniPrep2 is opened to the HTC reactor and IRMS and is observed for an unexpected rise in H_2_ signal above the EA background. (Note: an empty UniPrep2 carousel looks like an EA background regardless of the carousel temperature). The UniPrep2 background test applies to both CE and DVE approaches. Any residual moisture emanating from the carousel or the samples will be carried through the EA-HTC and raise the H_2_ background, thereby quantitatively revealing insufficient drying. This dryness check should be performed as follows:

• With the UniPrep2 “Isolation valve” closed, check the EA-IRMS H_2_ background in mV (e.g., approximately <200 mV).
• Start an IRMS time scan for H_2_ (e.g., 1200 s).
• Gently open the “Isolation valve” on the UniPrep2 to the EA-HTC reactor.
• Observe the H_2_ trace on the IRMS time scan. Usually, there are minor bumps or an H_2_ pulse due to the EA valve opening and helium pressure changes. Then the UniPrep2 background should decrease to approximately 120–200 mV and stabilize after approximately 10–15 min. If the H_2_ background is acceptably low (<200 mV) and stable beyond 30 min, it can be deduced that the samples are dried, and hydrogen-isotope analyses can proceed.
• However, if residual moisture is in the carousel or samples, the H_2_ background will rise (e.g., >500–1000 mV). It may rise well above 1000 mV, then slowly drop over time as H_2_O is carried from the carousel through the reactor. In this case, additional vacuum and helium flushing is required to dry the samples and carousel (e.g., repeat from Step 7).

Experience has shown that insufficient evacuation and helium flush times after sample vapour equilibration will lead to considerable H_2_O background as residual moisture remains in the samples. For example, after only 10 min evacuation pumping and 15 min of helium flushing on a carousel of keratin samples, opening the carousel to the EA reactor led to a rise in the UniPrep2 background on the IRMS to >2000–3000 mV – clearly, there was still considerable residual moisture still to be removed. After 3 h of helium flushing, the carousel was stable at UniPrepUniPrep2 background of approximately 120–160 mV. Importantly, it is likely that evacuation and helium flushing-time procedures will be sample-matrix specific. Hence, the researcher must determine the helium drying times for each organic sample matrix type.

The procedure to determine the helium flush time needed to achieve dried samples is as follows: load the UniPrep2 carousel starting with a set of silver microtube reference waters (e.g., VSMOW, GISP) and then several sequences of triplicates of one matrix equivalent unknown sample. After vapour equilibration of the entire set of samples (be sure to use a vapour depleted or enriched vapour ^2^H that is not close to that of the sample), and after 30 min of evacuation, helium flush the carousel for 1 h, then analyse the first triplicate set, then flush the carousel with helium for another hour, analyse the second triplicate set, and so on spanning for as many hours as needed. The *δ*^2^H values of the samples should reach an asymptote after a few hours or more, revealing the minimum online drying time. The samples are deemed thoroughly dried if there are no further changes in the *δ*^2^H values of the sample over time and helium flushing. This *δ*^2^H asymptote in hours (and perhaps adding 0.5 h as a precaution) should be applied to samples of that matrix type. Hydrophilic organic samples are expected to take considerably longer to dry online, with helium flush times possibly exceeding 3–4 h compared to less complicated organics (e.g., keratin). With more UniPrep2 drying experience in various laboratories, it is expected that a standardized helium (or dry N_2_) drying schema could eventually be established for routine application to the most common organics.

Step 9. CF-IRMS Analyses

With the samples proven to be fully dried online and the UniPrep2 background stable, the samples are ready for *δ*^2^H analyses. Ensure the UniPrep2 to EA “Isolation valve” is open and the “Helium In” and the “He purge” valves are fully closed. Leave the UniPrep2 temperature set to the operational setpoint. Initiate the analysis sequence from the CF-IRMS data system as per manufacturer software. Typically, following several H_2_ reference gas pulses and using a 5–10 s sample drop time, the sample H_2_ peak will appear after approximately 100–120 s (CF-IRMS and EA specific). With the chromium reactor configuration, and if fitted with a TCD, a small N_2_ peak may follow the H_2_ peak for nitrogenous samples on the TCD trace. With the carbon reactor configuration, a variable-sized N_2_ and a significant CO peak appear on the TCD after approximately 300–500 s. Again, all these timings are CF-IRMS and EA-specific and should be adjusted to ensure a complete return to EA baseline between samples. The H_2_ run should be checked for H_2_ retention time, peak shape, and peak area. Care should be taken that the EA reactor does not plug up with silver capsules, which generally leads to H_2_ peak broadening and an increase in retention time. Process the samples, standards, and controls as per manufacturer IRMS software or LIMS for Light Stable Isotopes (https://water.usgs.gov/water-resources/software/RSIL-LIMS/), and process the samples to report their final *δ*^2^H values on the VSMOW-SLAP scale.

Step 10. *δ*^2^H_n_ Calculations

### DVE approach

With the DVE approach, vapour equilibration of samples using two isotopically differing waters allows for the determination of the fraction of exchangeable hydrogen (ƒ_ex_) of the organic unknowns, the mass fraction hydrogen, and the *δ*^2^H_n_. The ƒ_ex_ result is obtained from measured *δ*^2^H isotopic difference in the unknown organic samples after equilibration with each of the two known and widely differing water vapours. For example, two vapours having *δ*^2^H values of +1.83 and −397.7 ‰ (VSMOW) were equilibrated with one unknown organic keratin sample. Using these two waters, the measured  *δ*^2^H_VSMOW_ values of the organic sample after each isothermal vapour equilibration were −80.0 ‰ and −91.6 ‰ (VSMOW), respectively. To determine the fraction of exchangeable hydrogen:

ƒ_ex_ = (*δ*^2^H_std‐EW_ – *δ*^2^H_std−DW_) / (*δ*^2^H_w‐EW_ – *δ*^2^H_w‐DW_)

ƒ_ex_ = (10.63 ‰ / 399.5 ‰) = 0.027 (or 2.7 % exchangeable hydrogen)

The *δ*^2^H_n_ result is determined using either the high *δ* or lower *δ* vapour-equilibrated sample after an accurate ƒ_ex_ has been determined:

*δ*^2^H_n_ = (*δ*^2^H_sample_ - (ƒ_ex_ * (*δ*^2^H_w‐EW_ – *δ*^2^H_w‐DW_)) / (1 - ƒ_ex_)

*δ*^2^H_n_ = (−91.6 – (0.027 * 399.5) / (1 – 0.027) = −83.3 ‰ (VSMOW) where *δ*^2^H and EW and DW subscripts refer to the measured organic *δ*^2^H results (normalized to VSMOW-SLAP scale) for the ^2^H-enriched and ^2^H-depleted value vapour exposures. In previous publications, the *δ*^2^H_n_ determination might have included an extra hydrogen-isotope fractionation factor (ε_sample-vapour_). However, this isotope-fractionation factor can only be determined if organic samples can be chemically treated to remove the exchangeable hydrogen fraction (e.g., nitration of chitin) and remeasured for the *δ*^2^H difference after vapour equilibration. Because most complex organic samples cannot be chemically treated by nitration, it is recommended to deprecate the incorporation of a temperature-dependant vapour-sample hydrogen-isotope fractionation factor for most organics [39].

The determined ƒ_ex_ is crucial because it controls the outcome and the accuracy of *δ*^2^H_n._ If a sample has no exchangeable hydrogen, ƒ_ex_ should be zero (e.g., no response from exposure to ^2^H-enriched or ^2^H-depleted vapours). If an organic sample is dried, ƒ_ex_ should produce the same value regardless of the isotopic composition of the two equilibration vapours used. However, suppose the ƒ_ex_ of a sample differs when using a separate set of ^2^H-enriched or ^2^H-depleted vapours. In that case, this could result from incomplete drying and lingering residual moisture in the samples (or the carousel). Quantifiable drying using the IRMS as the detector ensures that residual moisture is not a confounding factor (e.g., does ƒ_ex_ decrease or stabilize with more prolonged helium flushing and drying times?) and accordingly could lead to *δ*^2^H_n_ differences produced amongst laboratories if not repeatable. The ƒ_ex_ may be an additional useful diagnostic feature of the organic sample type in addition to its *δ*^2^H_n_ value. Generally, fresh organic matter and carbohydrates have higher ƒ_ex_ values, whereas refractory organic matter usually has a lower ƒ_ex_.

The elemental H mass fraction of the organic samples should also be determined and reported after CF-IRMS analysis. The mass % of H must be closely monitored with the DVE approach because this assay is also impacted by residual moisture that is measured as part of the total hydrogen. The elemental hydrogen mass fraction can be determined for unknown samples by (i) including accurately weighed samples of non-exchangeable H reference materials in each run with established elemental hydrogen concentrations (e.g., water in silver microtubes, USGS77, or NBS22a) and( ii) comparing their H_2_ peak areas and hydrogen mass fractions to the sample peak areas and thereby calculating the fraction of hydrogen of the samples using either the CF-IRMS H_2_ peak areas in LIMS for Light Stable Isotopes, or, if an EA TCD option is available using the H_2_ peak areas and conducting offline calculations. The elemental hydrogen composition of an organic sample should be stable and consistent after online drying. For example, suppose an organic reference sample (e.g., a USGS wood RM) with a certified 6 % total hydrogen mass fraction gives a determination with a higher value (e.g., 8 %). In that case, it is likely caused by residual moisture or from sample weighing errors. Careful drying and accurate sample and reference weighing are critical for mass fraction determinations.

### CE approach

Using the CE approach, the determination of *δ*^2^H_n_ uses a two-point isotopic normalization using the *δ*^2^H_n_ organic reference materials having consensus values on the VSMOW-SLAP scale. Single-point normalization is not recommended due to IRMS-specific scale expansion or contraction. Because so few *δ*^2^H_n_ working references exist, a three-point or more-point scale normalization is unlikely to be available.

In this example, a two-point normalization [40] of an unknown hair keratin sample (*δ*^2^H_n sample_) is done using the CBS (*δ*^2^H_n_ = −157 ‰ VSMOW) and KHS (*δ*^2^H_n_ = −35.3 ‰ VSMOW) keratin standard references [14]:

*δ*^2^H_n hair_ = *δ*^2^H_keratin std low VSMOW_ + (*δ*^2^H_hair meas -_ *δ*^2^H_keratin std low meas_) x ((*δ*^2^H_keratin std high VSMOW -_

*δ*^2^H_keratin std low VSMOW_) / (*δ*^2^H_keratin std high meas -_*δ*^2^H_keratin std low meas_))

In the laboratory, the uncorrected *δ*^2^H values for CBS and KHS from the CF-IRMS against H_2_ tank reference gas were +53.7 ‰ and +203.9 ‰ for CBS and KHS, respectively. The measured *δ*^2^H IRMS value for the hair sample was +99.3 ‰. Using the above equation, one can determine the *δ*^2^H_n_ of the hair sample (and those of others in the run):

*δ*^2^H_n hair_ = −157.0 + (99.3 - 53.7) x ((−35.3 - (−157)) / (203.9 – 53.7)) = −120.1 ‰ VSMOW

The 2-point normalization converts the uncorrected hair *δ-*value to *δ*^2^H_n_ on the VSMOW-SLAP scale. Notably, this normalization does not include other pre-processing data corrections that could be applied to improve the precision of the results, like ion source linearity corrections to correct for a predictable isotopic variance from sample weighing or time-linear instrumental drift, which are both readily determined and applied using data processing software like *LIMS for Light Stable Isotopes*.

Caution should be used when quoting *δ*^2^H_n_ values for organic working standards from older literature or non-authoritative sources. The status of organic *δ*^2^H_n_ reference materials is still unsettled, and the *δ*-values of some organic standards have changed considerably over time as hydrogen-isotope analysis methods. The recognition of the impact of exchange and drying has also evolved. Practitioners should be vigilant to the *δ*-values used by the laboratory for its organic references and report these in their publications. Only with full disclosure of operational conditions and assigned values can the results be used by others for comparative studies, for example, in an extensive exercise in the recalibration of hydrogen-isotope results obtained from multiple laboratories over several decades [41].

### Finishing and cleaning up

After the organic hydrogen samples have been successfully analysed using the UniPrep2, close the “Isolation valve" to the EA and CF-IRMS, turn off the UniPrep2 power, and let the carousel cool to room temperature. As required, close the EA to the CF-IRMS system on the mass spectrometer software. Ensure the “Vacuum Out” and “He in” valves are closed. Open the “He purge” valve on the top of the carousel (two counterclockwise rotations to vent) and allow the carousel to release its pressurized helium contents to atmospheric pressure as indicated by the Pressure gauge, and then firmly close the top “He purge” valve again. Loosen the carousel wingnuts and open the hinged cover plate. Check each sample well for silver capsules that may have gotten stuck and for signs of spilled sample powder or particle residues that could contaminate subsequent analyses. The carousel can be cleaned by wiping the top with a lint-free cloth and the sample wells by gently blowing them with compressed canned air. Loosen and reset the sample counter ring to the correct zero position. Depending on the number of samples and the amount of silver buildup, it may be needed to cool the EA, stop the helium flow, and remove and clean out the reactor of the silver capsule buildup. Unscrew and detach the UniPrep2 adaptor from the EA flange and then gently move it to the side or back to allow reactor removal, and take care not to damage the helium carrier gas lines or fittings. Follow the EA manufacturer instructions for HTC reactor removal and reinstallation. With a newly cleaned reactor in place, firmly re-attach the UniPrep2 to the EA reactor flange in readiness for equilibrating and running the next set of samples. It is recommended to close and evacuate the UniPrep2 carousel when not being used to avoid external dust from getting into the sample wells.

### Method validation

Given the lack of diverse organic reference materials for *δ*^2^H_n_ values, method validation is only possible using several USGS organic reference materials, for example, using the CBS and KHS keratins as the high and low bracketed calibration standards (either CE or DVE as described above) and running the USGS42 and USGS43 hair keratins as unknowns. Either approach should return the USGS-certified hair VSMOW *δ* values, as demonstrated in [14]. As more and more matrix diverse *δ*^2^H_n_ reference materials eventually become available, standard operating procedures (SOP) validation and interlaboratory replication are envisioned to play a vital role in advancing hydrogen-isotope analysis for different organic environmental matrices and studies.

## Ethics statements

*No ethical considerations were required.*

## CRediT authorship contribution statement

**Leonard I. Wassenaar:** Conceptualization, Methodology, Writing – original draft, Validation. **Leonardo Sisti:** Conceptualization, Visualization, Writing – review & editing. **Matthias Pilecky:** Writing – review & editing. **Martin Kainz:** Resources, Writing – review & editing.

## Declaration of Competing Interest

The authors declare that they have no known competing monetary interests or personal relationships that could have appeared to influence the work reported in this paper.

## Data Availability

No data was used for the research described in the article.

## Appendix Supplementary materials

Supplementary material and/or additional information [OPTIONAL] Supplementary material: Excel files with UniPrep2 analysis templates and example data processing are provided. Image, application 1
